# Main Factors Affecting Physicians’ Prescribing Decisions: The Iranian Experience

**Published:** 2018

**Authors:** Sayed Hesam aldin Sharifnia, Mehdi Mohammadzadeh, Gelareh Arzani, Jamshid Salamzadeh, Sayed Abolfazl Abolfazli, Alireza Zali, Ali Reza Khoshdel

**Affiliations:** a *Department of Pharmacoeconomy & Administrative Pharmacy, School of Pharmacy, Shahid Beheshti University of Medical Sciences, Tehran, Iran. *; b *Mofid Children’s Hospital, Shahid Beheshti University of Medical Sciences, Tehran, Iran. *; c *Department of Clinical Pharmacy, School of Pharmacy, Shahid Beheshti University of Medical Sciences, Tehran, Iran.*; d *Functional Neurosurgery Research Center, Shahid Beheshti University of Medical Sciences, Tehran, Iran.*; e *Faculty of Medicine, AJA University of Medical Sciences, Tehran, Iran.*

**Keywords:** Prescription behavior, Pharmaceutical Marketing; Consumer, Payers’ factors, Products’ factors, Environmental factors

## Abstract

Prescription decision making is a complicated phenomenon influenced by many factors including drug strength, the patient’s context, prescriber characteristics, health facilities, payment type, and pharmaceutical marketing. To evaluate the associations between each influenced factor and drug prescription method of Iranian physicians, we conducted an exploratory research, utilizing a questionnaire as quantitative research instrument. A sample of 460 physicians was asked to fill out the questionnaire, yielding 84% response rate. The statistical analysis from the collected data demonstrated that Iranian physicians mostly paid attention to the payment type, the patients’ individual factors and the products’ characteristics while prescribing a medicine. In addition, it was revealed that marketing expenditures did not have a high influence on the physicians’ demand for pharmaceutical products in Iran. The obtained results may be useful for Iranian pharmaceutical companies’ marketing strategy planners as well as the patients who are the exact consumers of the prescribed medicines.

## Introduction

There is an ever-growing interest shown in studying physicians’ decision making and the factors affecting it, lately. The primary reason is that physician valuating consumption of more than 10 percent of gross domestic product (GDP) of most developed nations to healthcare is highly integrated to pharmaceuticals. The global market of pharmaceutics is large, growing, and competitive and the immense significance of Pharmaceutical Companies in a country›s economy is evident. Indeed, that is the reason why Pharmaceutical companies are among the most evaluated and analyzed organizations in business today. 

However, the utilized marketing strategies in pharmaceutical industries are hugely different from those employed in other markets. Unlike the traditional buying decision process, the influencers, gatekeepers and deciders in the prescription medicine market are physicians and the patients are the buyers and consumers (4). Earlier studies statistically illustrated the significant positive effects of detailing and free drug samples as the two central components of pharmaceutical marketing practices on the number of new prescriptions issued by physicians (5). Other studies on pharmaceutical marketing also identified the key role of other marketing tools such as the relationship of pharmaceutical sales representatives with doctors, advertising, marketing research, public relations, and distribution on physicians’ prescription behavior (4, 6). Furthermore, a very important phenomenon in prescription behavior which should not be neglected is the continuity in prescribing the same company’s drugs defined as prescription loyalty which could be derived from positive experiences gained by a physician during the treatment phase after repeated prescriptions (7). Aside from marketing factors (advertising, sales representatives, drug expenses, and trade fairs) and professional factors (journals, prescription loyalty, opinion leader influence, and recommendations by colleagues), factors regarding patient characteristics, patient treatment history, comorbidity and payer type have obvious effects on physician choice behavior, as well. Due to the serious consequences and side-effects that might be made by prescribing the wrong products, physicians follow some treatment guideline focused on the importance of patient comorbidity and inherent characteristics such as age, gender, and race to choose the best available treatment for the patients (8). 

Taking all the above factors into account, it can be estimated that physician’s decision making on prescribing medicines has components of both consumer and industrial type. Therefore, it is a hybrid buying situation. Although the decision on which medicine will be prescribed should primarily depend upon scientific criteria, personal and social values as well as economic factors may play a role, especially when it is about diseases that may be treated by a few alternative medicines with negligible differences. Regarding the fact that supply of non-prescription drugs is tightly restricted by Iranian Food and Drug Organization (IR-FDO), equivalent to the American Food and Drug Administration (FDA), the huge importance of prescribed drugs in Iranian pharmaceutical market is inevitable. Thus, this present research attempts to empirically identify the major factors that influence physicians’ behavior in prescribing medicine to provide a holistic picture of drug prescription and expand the horizons of existing research in physician prescribing behavior in Iran. Hopefully, the weight of each influenced factors illuminated from this study will help the Iranian pharmaceutical companies to work in emergent pharmaceutical markets and these responsiveness estimates can then be used to appropriately target physicians by allocating detailing resources across physicians more effectively.

*Conceptual Framework and Methods*

From the above literature review, it is evident that the phenomenon of prescription is diffused and so many factors, including the prescribers (physicians), the consumer (patient) and the payer (insurance, state or user), are involved in the process (10). In Iran, it is mostly the doctors who specify the medicines they prescribe. Consequently, the decision is not made neither with the patient nor with the payer. However, the prescribers will make their decisions based on the following factors:

- Patients’ influence (profile such as age, race, gender; co-morbidity and treatment history)
- Physicians’ experience and product valuation
- Products’ influence (safety, efficacy, side effect, cost)
- Marketing activity of pharmaceutical strategies (samples, details, advertising)
- Payers’ influence (insurance, managed care, …)
- Environmental factors (relationships with pharmacists and coworkers, seminars and congresses, access to medicine, work place, …)

In order to fully consider all the influence factors on the phenomenon of medical prescription, five theoretical constructs, containing patients’ related factors, products’ related factors, payers’ related factors, environmental factors and marketing and strategies of pharmaceutical companies, were developed within a framework.

**Figure 1:**
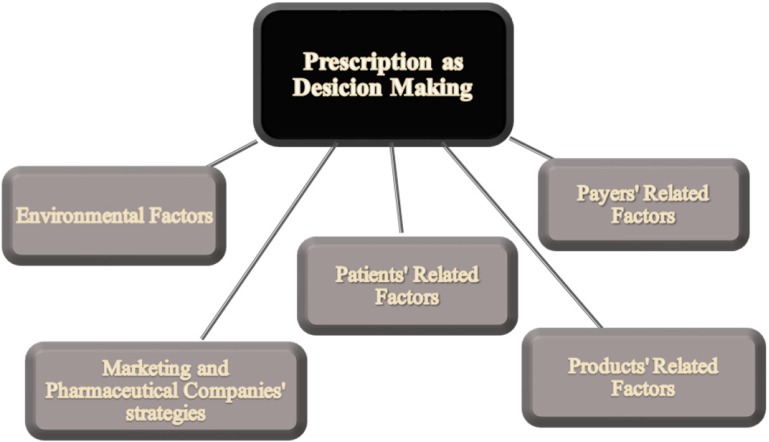
Influenced Factors on Prescription as Decision Making.

Subsequently, the following hypotheses are drawn based on the relationship between the physicians’ drug prescription behavior and the influenced factor discussed above:

- H1. Drug prescriptions are affected by the environmental factors
- H2. Drug prescriptions are affected by the products’ related factors
- H3. Drug prescriptions are affected by Marketing and Pharmaceutical Companies› strategies
- H4. Drug prescriptions are affected by the patients’ related factors
- H5. Drug prescriptions are affected by the payers’ related factors (Figure 1).

A quantitative approach with the survey method support was utilized to examine the five previously mentioned factors influencing prescribing decisions. A structured questionnaire adapted from modeling based on the researched theoretical references was made to collect data. The questionnaire was constructed on 63 questions in which 9 questions about demographics were nominal and ordinal scale and the other 54 questions were formulated in Likert scale, ranging from 1- strong agreement to 5- strong disagreement. The validity of the questionnaire was measured in a sub-group of ten Iranian doctors pursuing the aim that the content of the questionnaire could be correctly understood. Moreover, the reliability of the questionnaire, which examines the consistency of a concept measure, was assessed. The reliability of the questionnaire was broadly satisfactory as the Cronbach’s Alpha coefficient was totally exceeded 0.6 and some of them have reached to 0.9. 

In an attempt to select study respondents randomly from 39747 available Tehran’s physicians from all geographic regions, all specialties and all age groups, a two-stage cluster sampling technique was used. In the first stage, from 22 Tehran’s regions, 6 regions were randomly picked. Afterwards, in each region, 3 hospitals were chosen among the overall 84 hospitals available in these 6 regions.

Among 460 questionnaires distributed to all doctors working in the selected hospitals, 385 questionnaires were correctly answered which were further investigated statistically. Descriptive statistical analyses from the collected data were firstly performed. In addition, in this study, structural equation modeling (SEM) analysis using PLS with reflective indicators in Smart-PLS 2.0 was utilized for instrument validation and model testing.

*Analysis and Results*

*Descriptive analysis of demographic characteristics of participants*

Table 1 summed up the descriptive statistical analysis of the collected data from 385 participants. Based on the obtained results, the greater number of participants were females and between the age of 25 and 30, with the percentage of 53.6% and 46.4%, respectively. In terms of educational level, specialists account for the most common respondents (228 participants) with approximately twice the numbers of general practitioners and 5 times more than post-doctorate participants. A majority of the respondents (59.8%) had been engaged in the medical profession for one to five years and 58.6% of the participants tended to study less than 8 h a day. Interestingly, the participants were so hard-working that more than 74% of them worked more than 8 h a day. Among the possible work places available for the sample physicians, it could be seen that 48.3% of the doctors were associated with private clinics while 24.1% of them were affiliated with governmental hospitals.

*Descriptive analysis of research variables*

As illustrated in the Table 2, all participants agreed highly with the role of products’ factors in prescription as it showed a mean of around 4.1 out of 5 scale with a low standard deviation of 0.5. However, all five factors demonstrated as effective (means over 2 out of 5) with the differences distributed evenly as the standard deviation was relatively low in all the cases.

In terms of assessment of the normality of the data, which is a prerequisite for many statistical tests as normal data is an underlying assumption in parametric testing, Kolmogorov-Smirnov test was utilized. All five categories resulted in favorable normal hypotheses with the significance value above 0.05.

With respect to the reliability of each single concept revealed on table 3, the extracted mean variance and reliability obtained from each factors were above 0.5. In addition, in order to evaluate the reliability of the questionnaire, the composite reliability and Cronbach’s Alpha indexes were calculated. As both indexes were Figure out to be above 0.7, the reliability of the measuring instrument was proved. 

Table 4 summarized the inter-correlations and reliabilities of the study variable in the structural equation model. As is shown in Table 4, the reliabilities of all the variables, ranged from 0.87 to 0.71, are quite acceptable for research purposes.

Goodness of fit (GOF) index was also assessed revealing the discrepancy between the statistical model and the data at hand. As the obtained GOF index (0.646) was over 0.5, all models fitted satisfactorily and the main hypotheses of the research was thus confirmed.

Analysis of the overall coefficient of determination showed adequate fit indices following limits propose by Mulaik *et al*. (10). Higher than 0.5 coefficient index represents the efficacy of the study’s hypothesis and in this study it shows that 77% of changes in doctor’s decision making during drug prescription could be explained by the study’s five variables. 

## Results and Discussion

The theoretical models were estimated by t-value and the standardized coefficients (β) of the structural equation modeling. As summarized in Table 5, t-value and β obtained for products’ related factors, patients’ related factors and payers’ related factors would be counted as meaningful either if *P* < 0.05 or *P* < 0.01. In the lights of the findings of this studies summarized in the integrated model, products’ related factors, patients’ related factors and payers’ related factors have got the most influence on drug prescription of the Iranian doctors. 

H1. Drug prescriptions are impacted by the environmental factors.

As is shown in Table 5, t-value for this hypothesis was 0.797 which was not in the acceptable range of *P* < 0.05 (|t |> 1.96). As a result, the hypothesis is rejected with 95% of certainty as it is not statistically meaningful. 

**Table.1:** Descriptive Analysis of Demographic Characteristics

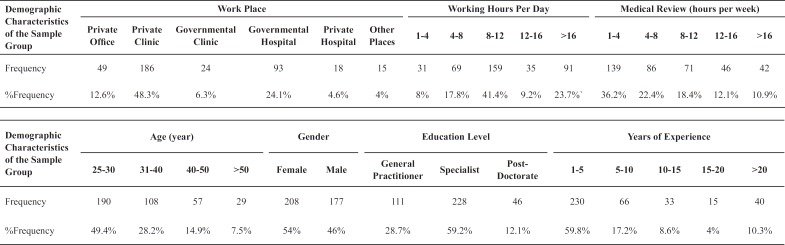

**Table.2:** Descriptive Analysis of Research Variables and Kolmogorov-Smirnov Test Result

**FFResearch** ** Variables**	**Mean** *	**Median** *	**Standard Deviation** *	**Quarter**			**Z-value**	**The Significance Level**	**Normality Result**
				First	Second	Third			
Patients Related Factors	3.644	3.667	0.633	3.200	3.667	4.025	0.651	0.79	Normal
Products’ Related Factors	4.083	4.059	0.522	3.765	4.059	4.471	0.930	0.353	Normal
Marketing and Pharmaceutical Companies’ Strategies	2.252	2.233	0.685	1.733	2.233	2.667	1.099	0.179	Normal
Environmental Factors	2.619	2.571	0.754	2.000	2.571	3.000	1.001	0.171	Normal
Payers’ Related Factors	3.316	3.200	0.889	3.000	3.200	4.000	0.540	0.933	Normal

* Sample Size = 385

**Table.3:** Reliability Indexes of the Study

**Latent Varient**	**AVE**	**Composite reliability**	**Coefficient of Determination**	**Cronbach’s Alpha**	$\sqrt[{2}]{\mathrm{AVE}}$	$\sqrt[{2}]{R^{2}}$	**GOF** *
Prescription as decision making	0.773	0.766	0.77	0.662	0.784	0.824	0.646
Environmental factors	0.512	0.826		0.762			
Products’ related factors	0.529	0.947		0.940			
Marketing and Pharmaceutical companies’ strategies	0.540	0.915		0.901			
Patients’ related factors	0.610	0.939		0.925			
Payers’ related factors	0.725	0.929		0.905			

* *GOF = Goodness of Fit*

**Table.4:** Intercorrelations among the Measured Variables.

**Latent Varient**	**1**	**2**	**3**	**4**	**5**	**6**
Prescription as decision making	0.879					
Environmental factors	0.571	0.715				
Products’ related factors	0.580	0.478	0.728			
Marketing and Pharmaceutical companies’ strategies	0.704	0.393	0.515	0.735		
Patients’ related factors	0.595	0.473	0.548	0.378	0.781	
Payers’ related factors	0.397	0.248	0.383	0.223	0.388	0.851

**Table.5:** Structural Equation Modeling Results.

**Dependent Variable**	**Independent Variable**	**β**	**t-value**	**Coefficient of Determination**	**Hypothesis Status**	**Relationship**
Prescription as decision making	Environmental factors (H1)	0.034	0.797	0.770	Rejected	Non reasonable
	Products’ related factors (H2)	0.167	3.284		Accepted	Direct
	Marketing and Pharmaceutical companies’ strategies (H3)	0.085	1.043		Rejected	Non reasonable
	Patients’ related factors (H4)	0.351	5.614		Accepted	Direct
	Payers’ related factors (H5)	0.500	6.346		Accepted	Direct

|t|>1.96 Significant at

< 0.05; |t|> 2.58 Significant at P < 0.01

Our result was in contrast with previously studies which mentioned the direct role of the information source and evidence in prescribing decisions. Smith *et al*., in one of the most significant volumes on pharmaceutical marketing qualifies their approach to marketing as environmentalist, thus highlighting the importance of environmental factors in marketing in pharmaceutical industry. He came to the conclusion that modern marketing is value-driven, where the consumers and other constituents of the environment are regarded as partners, and marketing itself is focused on creating and maintaining long-term relationships with the target environment, surpassing a relationship based on a simple transaction (11). The impact of social interactions and peer effects in the context of physician prescription choices was also studied by Nair *et al*. (12). Normally, most physicians attend workshops, seminars, and conferences where they are advised to prescribe a particular company’s drugs. The physicians also meet their peers and interact with them about their experiences. Nair *et al*. explained that the behavior of active research specialists, or “opinion leaders,” in the physician’s reference group had a significant influence on the physicians’ prescription behavior. Moreover, Al-Areefi *et al.* (13) mentioned the key role of medical representatives as the most common information source associated with the decision to prescribe newly marketed drugs, while patients, colleagues, and pharmacists also play a role in informing physicians about new or alternative drugs. Besides, the age of the physicians should also be taken into account. Cheraghali *et al*. (26) concluded that there is a substantial influence from gender of prescribers on prescribing pattern. The study showed that male prescribers are more restricted on rational prescription of injection. Therefore, it is suggested that in order to maximize effectiveness of corrective interventions, they should be tailored according to the gender of the prescribers.

The contrast observed in our study might be explained by the recent changes in medical regulations in Iran. Since 2013, the government has legislate a rule in healthcare system restricting the open pharmaceutical market with the aim of supporting the Iranian pharmaceutical companies, reducing the cost of healthcare services and monitoring the illegal imports of drugs and equipment. According to this legislation, all governmental hospitals have been required to substitute the prescribed pharmaceutical product to the cheapest available Iranian generic when the prescribing physician opposes it. If a physician vetoes the substitution for medical reasons, a committee will investigate the reasons and decide what changes should be considered. For the pharmaceutical products and equipment that does not have an Iranian substitution, there is a reference of confirmed brand-named products from which the governmental hospitals can decide to use. Obviously, one significant consequence of the recent legislation is reducing the number of pharmaceutical companies and available drugs. Besides, there is a rising competition between Iranian drug companies to grow in this available competitive pharmaceutical market by producing more high quality generic substitution to eliminate the needs of importing drugs. In addition, the huge role of medical representatives in providing commercial sources of information which influences the physicians’ prescribing behavior diminished to a large degree as the medical representatives could visit the physicians with difficulties and their products would not be purchased and substituted to the ones in use so easily.

H2. Drug prescriptions are impacted by the products’ related factors.

According to the data, this hypothesis was accepted due to the obtained t-value (3.284) which is higher than 1.96. As a conclusion, products’ related factors, such as its safety, efficacy, bioavailability, and so on were evaluated to have a meaningful and positive relationship with doctors’ prescription habits. It can be logically hypothesized that a physician will firstly take the product efficacy in to account in order to prescribe a medicine.

It can be obviously explained by the fact that a drug is an extremely tangible product and as such, the core and augmented constituents would be strongly studied (14). The core product reflects the medicine’s efficacy, usage, side effects, and contraindications. Likewise, the augmented constituent comprises a vast array of add-value services, i.e., the firm’s support with scientific evidence from clinical studies, the interaction and service from the firm’s detailer, in terms of other secondary add-value benefits (11, 15). An exploratory four-firm market-share study in 1996 found that the sales quantity of the pharmaceutical firm is hugely affected by the product quality (measured by efficacy, dosage forms, side-effect profiles, and indications for which the product had received FDA approval) (16). There is a significant level of corporate reputation for some corporate brands among all its stakeholders in pharmaceutical industry. Like some well-known companies, including Novartis, AstraZeneca, Sanofi, some Iranian pharmaceutical companies have tried to enhance their corporate brand equity through their marketing communications which can be resulted in corporate reputation as the extent to which the company is trusted, admired, and respected by physicians. If physicians assume that the company’s reputation is well deserved, they tend to always prescribe its drugs based on the company’s history of effective drugs (17). 

H3. Drug prescriptions are impacted by Marketing and Pharmaceutical Companies› strategies.

It can be seen from Table 5 that this hypothesis was also rejected showing a t-value of 1.043 which was lower than 1.96. So, Marketing and Pharmaceutical Companies› strategies factor did not have a reasonable relationship with doctors’ prescription behavior. These findings are in contrast with the well documented huge impacts of pharmaceutical marketing on educating and broadcasting of new medical treatments. 

For a pharmaceutical company, physicians’ and patients’ judgments or decisions about the company’s products defines as success. Having the aim of conveying integrated messages, pharmaceutical companies provide a vast array of services to all concerned parties utilizing various methods such as personal selling, which seems to be the most powerful in many marketing studies (18, 14), free samples and gifts including financing for domestic and international conference participation, travel and accommodation, medical education, meals, and small gifts (19, 20), advertising (in journals to reach physicians, and in other media to reach consumer–called Direct-to-Consumer advertising), and coupons (both physician and patient coupons) (6). Although the assumption that physicians’ prescribing behavior is only based on the rewards offered by the pharmaceutical companies seems unreasonable, the rewards certainly help physicians to remember the company brands resulting in higher benefits to the company (19). Gonul *et al*. described the anecdotal evidence from physician discussions that even with similar main ingredients in competitive brands, physicians avoid prescribing another brand for refills in order to keep the possible placebo effects of the original brand which has already worked for the patient. As a result, higher consumption of the companies’ products would be resulted (20).

However, the contrast findings from this study might be associated to the new Iranian governmental legislation in healthcare system. As previously mentioned, this legislation aims to diminish the healthcare expenses, widespread healthcare services, and strengthen Iranian pharmaceutical companies. Reaching these goals, high restrictions are provided to use the cheapest generic products or preferred drugs on the formulary, which are covered by insurances, in governmental hospitals. The formulary drugs are chosen regarding to their efficacy and characteristics after passing several steps and not just because of the medical representatives’ persuasive information to physicians. Due to the fact that amongst the products with equal quality and efficacy, the cheapest one is selected by governmental hospitals; pharmaceutical companies have to change their policies and cut their additional expenses to reduce their products’ total cost. In this way, their chance of election in the governmental hospitals’ formulary, which is extremely profitable for pharmaceutical companies, will be enhanced.

H4. Drug prescriptions are impacted by the patients’ related factors.

Based on the data estimated, t-value of this hypothesis was 5.614 which was markedly higher than 1.96. 

As a result, with 95% of certainty, the hypothesis was accepted. The relationship between the patients’ related factors and doctors’ prescription was counted as positive and meaningful. Showing a high β could indicate that this hypothesis was one of the most effective factor in doctors’ prescription. 

Patients are considered to be at the center of the prescribing process by physicians and the outcome of the treatment on patients’ health is the physicians’ main concern. Al-Areefi *et al*. reported that patient variables, including the patient’s clinical condition, failure of current therapy, financial situation and ability to purchase, and patient’s compliance, had powerful influence on the physicians’ prescribing decisions. Physicians will prescribe different medications regarding the severity of the patients’ conditions. For example, a more effective drug will be prescribed for patients in ICU service in comparison with other patients (13). Besides, physician’s price sensitivity may vary significantly regarding the patient income class. The physicians acting as agents for low-income patients and consider for lower price among medicines of similar efficacy for a given medical treatment. Likewise, patient’s psychological reaction to the product usage or efficacy may also mediate the physician’s prescribing behavior (14). Researches have shown that previous use of an effective pharmaceutical product promotes the prescribing of that product as refills. Unless available reports of some adverse side effects from the patients, the physicians’ usually do not tend to change the prescribed drugs. Besides, patients also often are not in favor of changing the medicines’ brand. It is mostly obvious in older patients and patients with chronic disease who have less favorable attitudes toward changing the brands even if the prescription is not covered in drug insurance (13, 21, 22). Thus, in terms of subjective knowledge, it could be interpreted that the more the doctors’ knowledge about the patients’ condition, the more satisfaction would be resulted by the advised medical prescription.

H5. Drug prescriptions are affected by the payers’ related factors

Payers’ related factor was also a hypothesis which was accepted with 95% of certainty according to the high t-value of 6.346. The hypothesis was actually evaluated as the most effective factor in drug prescription, regarding the highest β in comparison with all four other factors mentioned.

One of the distinctive characteristics of the healthcare systems is the existence of intermediate parties, such as governments, health maintenance organizations, or private insurers, which relates the price charged by the manufacturers and the quantity demanded by the patients (23). The presence of insurance assures the physicians to promote the choice of a more expensive treatment among alternatives with less concern in comparison to the patients who did not have insurance (24). Besides, previous researches have shown that pharmacists have an incentive to dispense the uninsured prescription with lower priced generic drugs as offering lower prices not only resulted in patients’ more satisfaction with the prescriber and the pharmacist, but also resulted in patients’ more compliance to the medication (25). 

In Iran, insurances provide the cost of the cheapest available generic drugs and equipment and Iranian pharmacists have required to substitute the prescribed medicine to the cheapest available generic when neither the prescribing physician nor the patient opposes it. Patients who oppose substitution have to pay the difference in price themselves. Regarding the fact that brand-name products are mostly not covered in insurances and their price is much higher than the cheapest generic drugs, patients show less desire to purchase brand-name products in comparison with the generic ones. As a result, the Iranian insurances help to strengthen the Iranian pharmaceutical companies by covering the price of the generic drugs instead of the brand-name pharmaceutical products. Moreover, the two significant roles of the payers on Iranians’ physicians could not be neglected. On the one hand, payers try to induce the physician to prescribe Iranian generic drugs which are cheaper or to prescribe the preferred drugs which are listed on the formulary for which the payers have a favorable contract with the manufacturer and on the other hand, payers have some control over the physician directly since they pay the physician fees and decide which physicians remain in or out of the preferred network. It should be noted that physicians tend to work with the insurances as to attract more patients. As a result, the vital role of the payer in health care system is not doubtable.

## Conclusion

This study integrated the different factors which are most significant determinants of decision making of the Iranian physicians, such as drug characteristics, environmental factors, pharmaceutical company strategies, insurances and patients’ individual factors. The findings of the study imply that environmental factors and pharmaceutical advertising have no effects of physicians’ prescription behavior, while the pharmaceutical products’ characteristics, the patients’ conditions and the insurances coverage play the vital roles in drug consumption. As many people pay attention to the expense of the medicine, physicians will be obliged to prescribe either cheap drugs or the drugs which are covered by insurances. Furthermore, the products’ pharmaceutical characteristics are as significant as the two mentioned affecting factors. A product with low efficacy, low bioavailability and pharmacokinetic characteristics will be omitted in the competitive environment of pharmaceutics and will lose the chance of being known and prescribed.

This study provides some data against the effects of environmental factors and the pharmaceutical marketing strategies on the overall drug prescription behavior in Iran. However, further research along these lines with more focus on dividing the health care professionals in various groups may be required to discuss the exact effects of these factors.
